# Modelling predicts differences in chimeric antigen receptor T-cell signalling due to biological variability

**DOI:** 10.1098/rsos.220137

**Published:** 2022-08-24

**Authors:** Vardges Tserunyan, Stacey D. Finley

**Affiliations:** ^1^ Department of Quantitative and Computational Biology, University of Southern California, Los Angeles, CA, USA; ^2^ Department of Biomedical Engineering, University of Southern California, Los Angeles, CA, USA; ^3^ Mork Family Department of Chemical Engineering and Materials Science, University of Southern California, Los Angeles, CA, USA

**Keywords:** constrained optimization, immunotherapy, CAR T cells, systems biology model

## Abstract

In recent decades, chimeric antigen receptors (CARs) have been successfully used to generate engineered T cells capable of recognizing and eliminating cancer cells. The structure of CARs typically includes costimulatory domains, which enhance the T-cell response upon antigen encounter. However, it is not fully known how those co-stimulatory domains influence cell activation in the presence of biological variability. In this work, we used mathematical modelling to elucidate how the inclusion of one such costimulatory molecule, CD28, impacts the response of a population of CAR T cells under different sources of variability. Particularly, we demonstrate that CD28-bearing CARs mediate a faster and more consistent population response under both target antigen variability and kinetic rate variability. Next, we identify kinetic parameters that have the most impact on cell response time. Finally, based on our findings, we propose that enhancing the catalytic activity of lymphocyte-specific protein tyrosine kinase can result in drastically reduced and more consistent response times among heterogeneous CAR T-cell populations.

## 1. Introduction

T cells engineered to express a chimeric antigen receptor (CAR) have emerged as a novel tool for combatting cancer by generating an immune response against cancer cells. The key step in this immunotherapeutic approach is to produce T cells expressing an artificially designed CAR, which activates the T cell upon encountering a target cancer cell [1]. To achieve this goal, CARs feature an antigen recognition domain derived from a single-chain variable fragment of a monoclonal antibody specific to the antigen of interest, while their cytoplasmic portion includes different combinations of intracellular signalling domains [2]. The first generation of CARs had a single cytoplasmic CD3*ζ* signalling domain attached to the recognition domain by a transmembrane linker. Later advances in CAR design resulted in second-generation CARs, which have an additional cosignalling domain originating from natively occurring costimulatory receptors [3]. The cytoplasmic domain of the CD28 costimulatory receptor is a commonly used example [4]. Notably, adding CD28 cosignalling enhances proliferation and target cytotoxicity among the CAR T cells [5]. In spite of significant progress, certain gaps in CAR T-cell performance remain. Importantly, current CAR T-cell therapies are sufficiently effective only against liquid tumours, while the performance against solid tumours is still limited [6]. In addition, CD28-based CAR T-cell therapies suffer from some major side effects [7], which can be life-threatening [8]. These considerations necessitate further improvements in the CAR T-cell technology, with two broad aims: on one hand, make CAR T cells efficient against a more extensive set of targets; on the other hand, mitigate the observed side effects.

Mathematical modelling is an integral approach in systems biology and has been applied to explain biological phenomena and generate testable hypotheses to guide experimental research [9]. One successful strategy of mathematical modelling is ordinary differential equation (ODE)-based mechanistic modelling of biochemical processes [10]. With suitable estimates of interaction parameters and initial concentrations, these ODEs can be integrated to obtain time courses for each component of the system. In the past, various research groups have used ODE-based mechanistic modelling to gain quantitative insights into immune cell signalling dynamics. For example, this approach was successful in describing native T-cell receptor-induced activation of mitogen-activated protein kinase/extracellular-regulated kinase (MAPK/ERK) signalling [11]. Our group has used this approach to simulate the dynamics of ERK signalling in CAR T cells in response to antigen binding [12]. Particularly, by calibrating model parameters against data obtained by phosphoproteomic measurements, it was possible to obtain a close correspondence between model predictions and observed experimental dynamics. This was used to make quantitative comparisons between first-generation CAR T cells with CD3*ζ* as the only signalling domain and second-generation CAR T cells with an additional CD28 co-stimulatory domain. Thus, modelling was successfully used to gain quantitative insights into CAR T cells and augment experimental knowledge.

Variability has been observed for essentially all dimensions of single-cell measurements, with the ensemble behaviour of a cell population not necessarily reflecting the behaviour of an individual cell [13]. Examples of variability caused by differential protein expression have been observed among endogenous T-cell populations, including T regulatory cells [14], T helper cells and T killer cells alike [15]. The influence of variable protein levels on the accuracy of the nuclear factor kappa-light-chain-enhancer of activated B cells (NF*κ*B) signal transduction pathway in CAR cells was subject of a detailed analysis by our research group [16]. Although we previously used our model of CAR-mediated signalling to investigate the effects of variability in the expression levels of signalling proteins on ERK activation [17], other manifestations of variability remain to be addressed. First, while in prior single-cell simulations, we assumed that the cell encounters a well-defined antigen concentration on the surface of the target, this is not true in the therapeutic setting. For example, prior measurements on primary myeloma cells found a range of values for the cell surface concentration of CD19 [18], the signature antigen targeted by the recognition domain of many CAR T cells. Second, the chemical reactions that mediate signal transduction are subject to fluctuations not only owing to stochastic protein expression, but also variability in cell state, local microenvironment and the number of molecular collisions [19,20]. Thus, in our current work, we set out to investigate and compare the performance of first-generation and CD28-bearing second-generation CAR T cells under two modes of heterogeneity: exposure to stochastic concentrations of the target antigen and stochastic kinetic rates of signalling processes. Then, we quantified the importance of different kinetic parameters for determining the ERK activation time of cells. Finally, based on these findings, we propose strategies to further enhance the efficiency of CAR T cells in a therapeutic setting.

## 2. Methods

### 2.1. Chimeric antigen-receptor-induced extracellular-regulated kinase signalling model

The ODE-based model used in our work was developed in Matlab by our research group [12]. The model includes four signalling modules: phosphorylation of immunoreceptor tyrosine-based activation motif (ITAM) regions of the CD3*ζ* domain in response to antigen binding, inhibitory activity of CD45 and SHP1, linker for activation of T cells (LAT) signalosome formation and MAPK signalling (electronic supplementary material, figure S1). The model was calibrated on experimental data and gives accurate quantitative estimates of the levels of signalling species, indicating that it constitutes a plausible description of the underlying biological processes. Particularly, the model gives a mechanistic explanation for the increased cytoplasmic concentration of doubly phosphorylated ERK (ppERK) in response to antigen binding to the CAR. We chose to use ppERK as the primary model output, as it mediates many cell responses accompanying activation. Given the non-transient ‘all-or-nothing' response typically displayed by ppERK in response to antigen stimulation [11], we termed all cells with more than half of their total ERK pool in the doubly phosphorylated form as ‘active', with the time needed to reach this state termed ‘activation time'.

### 2.2. Monte Carlo simulations

In simulations of variable antigen concentration encountered by CAR T cells, we assumed the antigen distribution (in units of molecules µm^−2^) to be lognormal with scale parameter *µ* = 1.0 and scatter parameter *σ* = 0.5. Our choice was based on the fact that most intracellular protein abundances closely obey a lognormal distribution [21]. We picked the scatter and scale parameters to match the observed concentration of CD19, a frequent target of CAR T-cell therapies, which was measured to be between 0.16 molecules µm^−2^ and 5.2 molecules µm^−2^ in primary myeloma cells [18]. Simulations for the variability of kinetic parameters were carried out by sampling each parameter from an independent normal distribution with the mean equal to the parameter's accepted value and a standard deviation equal to a third of the mean. These simulations were repeated for different antigen concentrations: 4.5 molecules µm^−2^ (‘low’, close to experimentally observed) and 45 molecules µm^−2^ (‘high', near saturating). Monte Carlo simulations were run in Matlab for 10^5^ iterations, since using this number showed a reliable convergence with multiple random seeds that we tested.

### 2.3. Gradient-boosted tree predictor

The gradient-boosted tree ensemble (GBTE) is a nonlinear machine learning method used successfully for both regression and classification tasks. It is based on a succession of individually weak decision trees. The first tree in the sequence fits the observed outcome directly, while each successive tree fits the residual left from the collective prediction of its predecessors. Thus, together, these decision trees achieve a significantly enhanced performance over a single decision tree [22]. We used the *scikit-learn* implementation of gradient-boosted trees in *Python 3.7* [23] to obtain a predictor of cell activation time based on the values of kinetic parameters as sole input. The synthetic dataset for training and testing was generated by using the ODE-based model to compute ERK activation while simultaneously varying values of 48 impactful kinetic parameters. These parameters were selected based on our prior findings, which showed that among all the kinetic parameters of the model, only this set has a measurable impact on ERK activation time [24]. We chose to simulate 10^5^ different values for the kinetic parameter vector since at this value, the accuracy of the predictor converged to a consistent number for all random seeds we tested. The accuracy of the GBTE was adjusted by tuning hyperparameters and evaluating resultant performance by fivefold cross validation of the coefficient of determination (*R*^2^) and explained variance (EV). Notably, by analysing the formulae for these metrics, it can be shown that if the obtained *R*^2^ and EV are equal, then the predictor is unbiased.

### 2.4. Permutation importance scores

The permutation feature importance score is defined to be the decrease in the *R*^2^ value of model prediction when a single feature value is randomly shuffled across all data points [23]. This shuffling procedure preserves the marginal distribution of the feature across the population, but otherwise decouples it from the output value of the data point. The underlying assumption is that if a feature is important in determining the output value, then this population-wide random shuffling will greatly reduce the predictive power of the model and result in a proportionate drop in predictor performance as evaluated by the *R*^2^ value. Similarly, since this decoupling procedure is highly unlikely to improve the performance of a predictor, permutation importance scores cannot be negative. We used the *scikit-learn* library in *Python 3.7* to calculate permutation importance scores with five different shufflings for each parameter, using these iterative calculations to evaluate the statistical significance of obtained scores according to a one-tailed *t-*test.

### 2.5. Parameter selection by optimization

Having quantified the importance of kinetic parameters on ERK activation time, we had the goal of isolating a handful of kinetic parameters whose manipulation would result in the largest reduction of ERK activation time. For this purpose, we picked as candidates the five parameters with the highest permutation importance score among first-generation cells in low-antigen conditions, excluding the affinity constant between the CAR and the antigen (to focus on targeting catalytic activities). Next, we used particle swarm optimization to minimize the following objective function:

$$T_{\mathrm{ERK}}+\;\lambda \sum \left|\log\frac{\;p_{i}}{\;p_{i,0}}\right|\;,$$

where *T*_ERK_ is ERK activation time, *p_i_* is the parameter value to be optimized, *p_i,0_* is the default value of that parameter and *λ* is a user-defined constraint parameter that specifies how much the ratio of the optimal and default parameter value is weighted. The structure of the objective function, inspired by the least absolute shrinkage and selection operator (LASSO) parameter selection technique, allows us to optimize parameters with the goal of decreasing activation time while changing as few parameters as possible. Particularly, it is long established that the penalization of the sum of absolute values in classic LASSO results in parameter selection by which only a subset of parameters is assigned non-zero values, while the rest remain at zero [25]. Since, in our case, the ‘default' value of the parameter is non-zero, the use of the logarithm of fold change allows us to assign zero penalty to unchanged values, while penalizing any change in proportion to the default value. We hypothesized that this manner of penalization would result in parameter selection such that ERK activation times would be reduced by manipulating as few parameters as possible. We varied *λ* starting from a large enough value (*λ* = 10) so that the constraint term is prohibitively large and prevents any change in parameter values, down to a low enough value (*λ* = 0.1) to identify parameters that are consistently selected by the optimization procedure.

## 3. Results

### 3.1. Population response with variable antigen exposure

**Figure 1.**
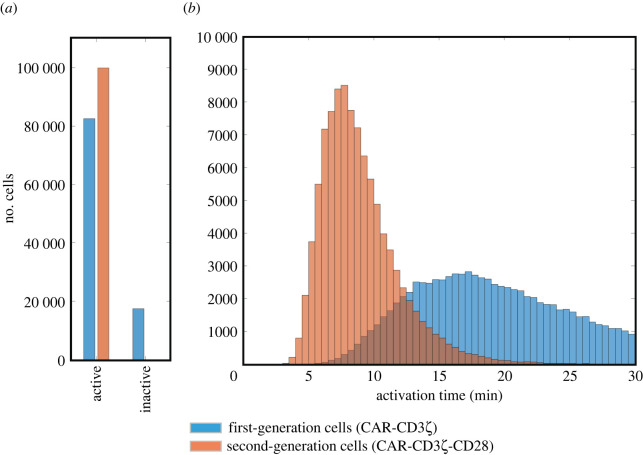
Activation of CAR T cells exposed to varying amounts of antigen. Predicted activation for 10^5^ cells with first- and second-generation CAR constructs stimulated by the same stochastic antigen concentrations *in silico*: (*a*) the number of cells becoming active or remaining inactive in the course of the 30 min simulation (no inactive cells were detected among second-generation cells) and (*b*) distribution of the activation times for cells that became active in the course of the 30 min simulation.

We simulated the response of CAR T-cell populations to a distribution of antigen concentrations. Thus, 10^5^ cells expressing either the first- or second-generation CARs were stimulated by an antigen concentration coming from a lognormal distribution *in silico*, and their activation times were recorded. Particularly, we predicted the number of CAR T cells that became active during the simulation (figure 1*a*) and summarized their activation times in a histogram (figure 1*b*). The presence of the CD28 costimulatory domain in the second-generation CAR resulted in a higher percentage of activated cells (82.6% versus 99.9% for first- and second-generation CAR T cells, respectively), with a shorter mean activation time (18.5 min versus 8.9 min for first- and second-generation cells, respectively). Additionally, the distribution of the population response has a smaller standard deviation among second-generation cells compared to the first-generation cells (3.0 min versus 5.4 min, respectively). This indicates that the CD28 domain not only confers greater activation in a shorter time, but also provides for a more consistent response. These effects of the CD28 domain can be attributed to the shifted dose response curve (electronic supplementary material, figure S2).

### 3.2. Population response with variable kinetic parameters

**Figure 2.**
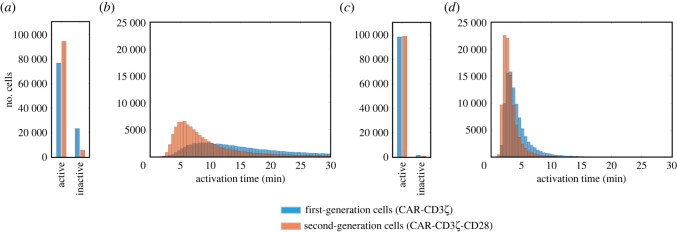
Activation of CAR T cells with varied kinetic parameters. Predicted activation for 10^5^ cells with first- or second-generation CAR constructs stimulated by the same antigen concentration (high or low) with stochastic kinetic parameters *in silico*: (*a*) the number of cells becoming active or remaining inactive in the course of the 30 min simulation with low-antigen stimulation, (*b*) distribution of the activation times for cells from (*a*) that became active in the course of the 30 min simulation, (*c*) the number of cells becoming active or remaining inactive in the course of the 30 min simulation with high-antigen stimulation, and (*d*) distribution of the activation times for cells from (*c*) which became active in the course of the 30 min simulation.

Another source of variability we set out to explore is the variability in effective rates of the reactions involved in signal transduction. Various sources of biological noise can result in heterogeneity of effective kinetic rates across a genetically uniform population. Thus, it is important to compare the performance of cells engineered with first- and second-generation CARs given variable kinetic parameters to account for the consequences of this mode of heterogeneity. To investigate the effects of kinetic variability, we performed simulations for first- and second-generation cells with randomized values of 48 influential parameters in conditions of either ‘low' or ‘high' antigen exposure. As evidenced by the resulting population distributions, the inclusion of the CD28 domain results in shorter mean activation time and a smaller standard deviation (figure 2). Particularly, in low-antigen conditions, more cells were activated in cells expressing the CAR that contains the CD28 domain (76.7% versus 94.2%, for first- and second-generation cells, respectively), with a shorter activation time (14.1 min versus 8.9 min, for first- and second-generation cells, respectively) and reduced standard deviation (6.5 min versus 5.2 min, for first- and second-generation cells, respectively). In high-antigen conditions, the presence of CD28 caused a similar change in the population response, albeit less pronounced (98.4% versus 98.9% of cells were activated, with mean activation time of 4.5 min versus 3.3 min and standard deviation of 2.8 min versus 2.0 min, for first- and second-generation cells, respectively).

### 3.3. Mechanism of CD28-induced changes in population behaviour

**Figure 3.**
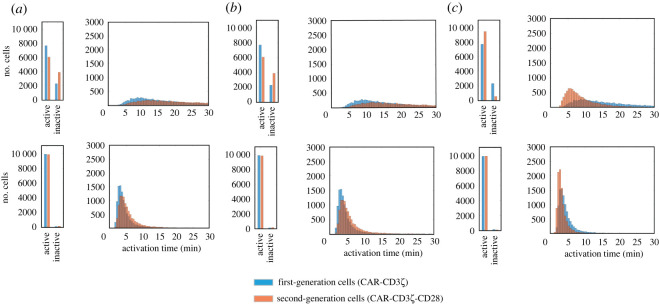
Activation of CAR T cells considering alternative CD28 signalling mechanisms. Predicted activation for 10^5^ cells with first- and second-generation constructs with one CD28 signalling mechanism implemented at a time. (*a*) CD28 exclusively associates with Grb2, (*b*) CD28 exclusively associates with GADS, and (*c*) CD28 exclusively affects the activity of LCK. Bar plot shows the number of cells becoming active or remaining inactive in the course of the 30 min simulation. Histogram shows the distribution of the activation times for cells that became active in the 30 min simulation. Top, low-antigen simulation. Bottom*,* high-antigen simulation.

A natural question to pursue is to determine the mechanism by which CD28 causes this reduction in the mean and standard deviation of response times. Specifically, we focused on three possible mechanisms: the interaction between CD28 and the adaptor protein Grb2, the interaction between CD28 and the adaptor protein GADS [26], and the enhancement of the catalytic activity of lymphocyte-specific protein tyrosine kinase (LCK) by CD28 [24]. The baseline model includes all three of these CD28 mechanisms (electronic supplementary material, figure S1). To isolate the contribution of each interaction, we again ran simulations with stochastic kinetic parameters but with only one CD28-mediated mechanism available at a time. We found that in the case of the isolated CD28/Grb2 interaction, second-generation cells perform poorly compared to first-generation cells, with increased mean activation time and standard deviation (figure 3*a* and table 1). Similar results were obtained for the case of the isolated CD28/GADS interaction (figure 3*b* and table 1). On the other hand, with the isolated effect of CD28 on LCK catalytic activity, we saw the reduced mean and standard deviation that are the hallmark of second-generation cells (figure 3*c* and table 1). Thus, based on our simulations, the kinetic effect of CD28 on LCK activity is the leading mechanism of CD28's role in influencing ERK activation. Overall, the interaction between CD28 and Grb2 or the interaction between CD28 and GADS proved insufficient to bring about any improvement, while CD28 on LCK's catalytic activity is shown to be necessary and sufficient.

**Table 1.** Effects of CD28 mechanisms on predicted cell activation.

condition	%-age cells active		mean activation time (s.d.), min	
	low antigen	high antigen	low antigen	high antigen
CD3ζ-only CAR	77.0	98.7	14.2 (6.5)	4.5 (2.8)
CD28/Grb2 interaction only	60.9	98.4	16.6 (6.6)	5.6 (3.5)
CD28/GADS only	60.9	98.4	16.6 (6.6)	5.6 (3.5)
CD28-mediated LCK effects only	94.3	99.1	9.1 (5.1)	3.4 (2.0)

### 3.4. Sensitivity analysis of activation time

Next, we aimed to quantify the impact of each parameter on ERK activation time through a data-driven procedure. In order to obtain importance scores for those 48 parameters, we first created a synthetic dataset in which the model was simulated for 10^5^ different and independently sampled values of the 48 parameters. This created a 48-by-10^5^ matrix of parameter values with corresponding activation times. The procedure was performed for four settings: first- and second-generation cells, each in ‘low' and ‘high’ antigen conditions. Then, we developed a GBTE and trained it on each dataset. Here, each of the 48 parameters was treated as an input feature and activation time as the output value. Hyperparameters were tuned until satisfactory predictive performance by fivefold cross-validation was obtained. Two performance metrics of the resulting GBTE, *R*^2^ and EV, are given for each condition in table 2. Since the obtained values for *R*^2^ and EV are identical in each setting, this implies that the GBTE is an unbiased estimator.

**Table 2.** Performance of the gradient-boosted tree ensemble on various datasets.

construct used: antigen conc.:	first-generation	second-generation
‘low'	*R*^2^ = 0.905 ± 0.0008	*R*^2^ = 0.900 ± 0.0004
	EV = 0.905 ± 0.0008	EV = 0.900 ± 0.0004
‘high’	*R*^2^ = 0.816 ± 0.0024	*R*^2^ = 0.807 ± 0.0035
	EV = 0.816 ± 0.0024	EV = 0.807 ± 0.0035

**Figure 4.**
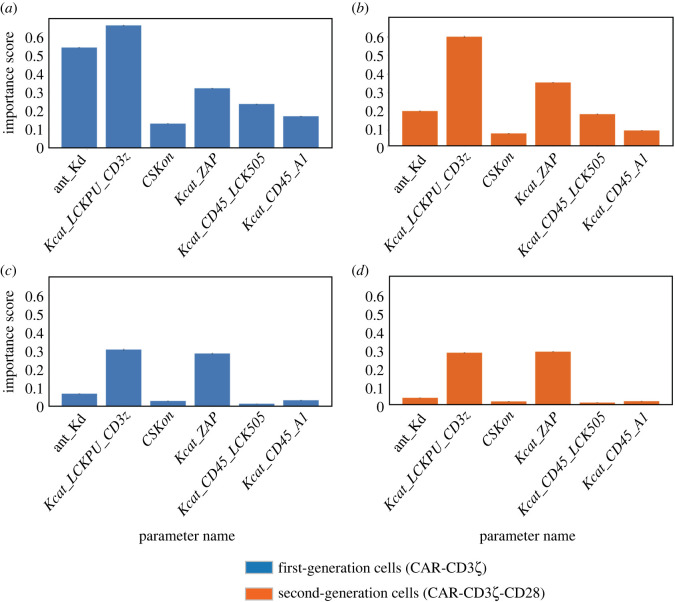
Permutation importance scores for select kinetic parameters used in machine learning model to predict cell activation times. A gradient-boosted tree was used to predict the cell activation times based on model kinetic parameters. We show the most important kinetic parameters that influence the predicted activation time under different conditions: (*a*) CAR-CD3*ζ* with low-antigen concentration, (*b*) CAR-CD3ζ-CD28 with low-antigen concentration, (*c*) CAR-CD3*ζ* with high-antigen concentration, and (*d*) CAR-CD3ζ-CD28 with high-antigen concentration.

With a successful predictor at hand, we set out to quantify the importance of each parameter for the prediction made by the GBTE. We did this by using permutation importance scores. The importance of each parameter was quantified in each of the four conditions (figure 4; electronic supplementary material, figure S3). With these results, we isolated the top five kinetic parameters that have a large impact on first-generation cells in low-antigen conditions: the catalytic activity of LCK in phosphorylating ITAM regions of CD3*ζ* (*Kcat_LCKPU_CD3z*), association rate of CSK with LCK (*CSKon*), catalytic activity of ZAP70 (*Kcat_ZAP*), catalytic activity of CD45 in dephosphorylating LCK (*Kcat_CD45_LCK505*) and the catalytic activity of CD45 in dephosphorylating ITAM regions of CD3*ζ* (*Kcat_CD45_A1*). Notably, the three highest-scoring parameters were identical between first- and second-generation cells in low-antigen conditions.

### 3.5. Parameter selection by constrained optimization

**Figure 5.**
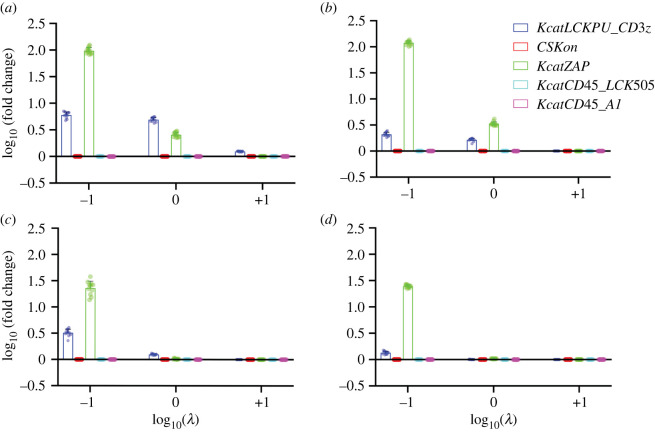
Results from constrained optimization. Logarithms of fold changes in each of the five parameters used in the constrained optimization to minimize activation time are shown for given constraint strength (*λ*), under different conditions: (*a*) first-generation cells under low antigen, (*b*) second-generation cells under low antigen, (*c*) first-generation cells under high antigen, and (*d*) second-generation cells under high antigen.

We hypothesized that owing to the large impact in determining the activation time of the cell, each of the five influential parameters identified from the GBTE could serve as a target for engineering more efficient CAR T-cell lineages. A key goal of such engineering would be to minimize the number of interventions into the system given the complexity of designing proteins with desired properties and then expressing them in engineered cells. Thus, we performed parameter selection by constrained optimization to identify which one of these parameters could serve as the most optimal target of a limited experimental intervention. The objective function was designed to minimize activation time while modifying as few parameters as possible (see Methods section). This optimization routine was performed with both first- and second-generation cells, each in low- and high-antigen conditions, and with different values of the optimization constraint parameter (figure 5). When the value of the constraint parameter is 10, kinetic parameters do not change at all in course of the optimization. However, as we relax the constraint parameter (i.e. reduce its value to 1.0), two kinetic parameters change within one order of magnitude to actuate a decrease in cell activation time: *Kcat_LCKPU_CD3z* and *Kcat_ZAP*. These two kinetic parameters remain the only ones whose values should be optimized even when we further decrease the constraint parameter. Thus, we predict that when the goal is to decrease cell activation time, an increase in *Kcat_LCKPU_CD3z* and *Kcat_ZAP* will result in the largest such decrease.

**Figure 6.**
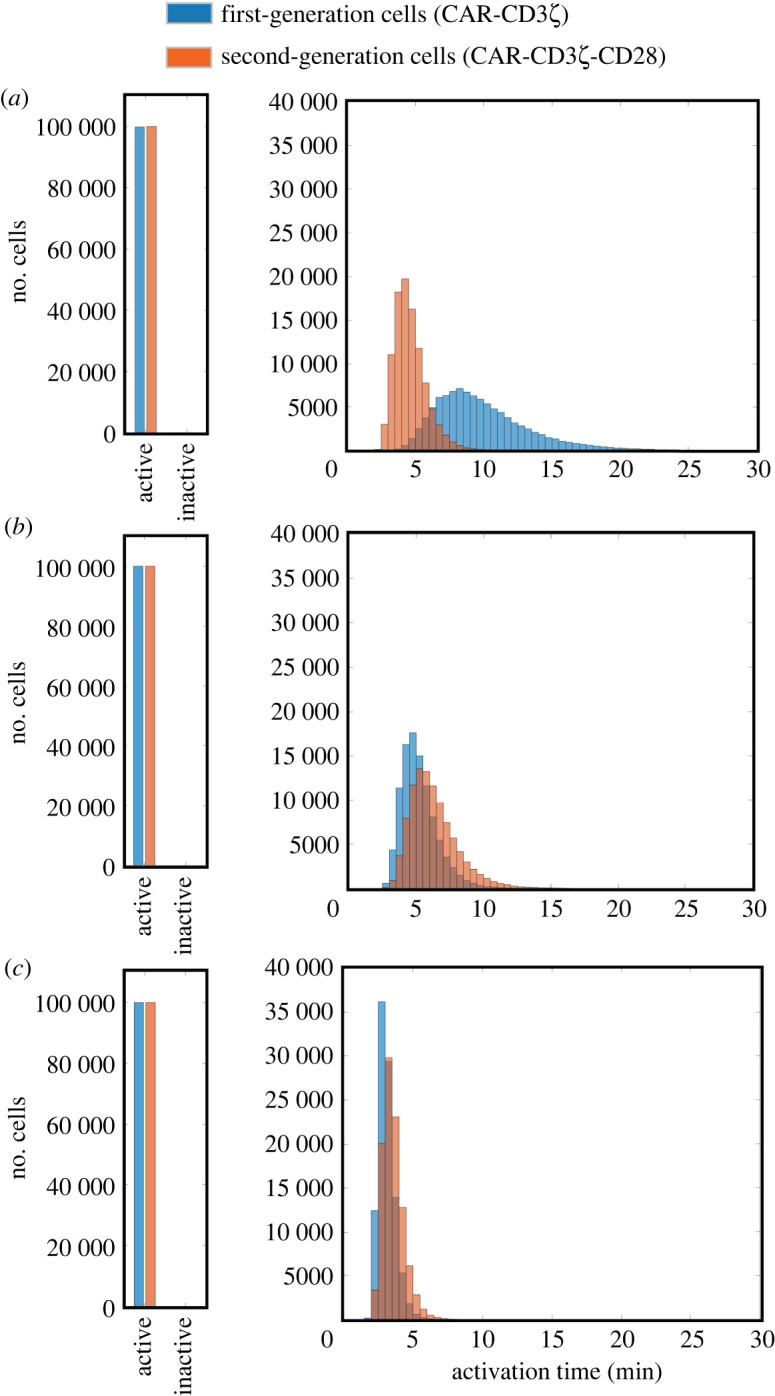
Activation of CAR T cells with optimized parameter values. We simulated simulation of cells with first- or second-generation CAR constructs upon implementing the optimized values of the two most influential parameters, *Kcat_ZAP* and *Kcat_LCKPU_CD3z*. (*a*) Simulated results with optimized *Kcat_ZAP* only, (*b*) simulated results with optimized *Kcat_LCKPU_CD3z* only, and (*c*) simulated results with both *Kcat_ZAP* and *Kcat_LCKPU_CD3z* optimized.

**Figure 7.**
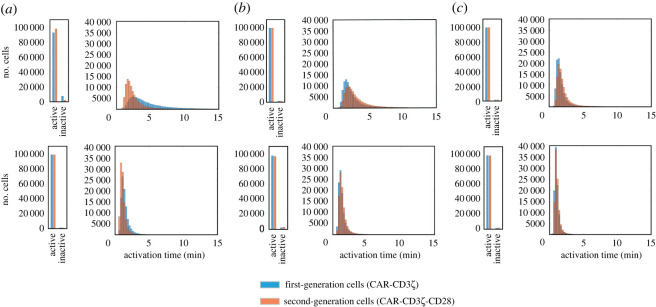
Activation of CAR T cells with varying kinetic parameters and optimized values of *Kcat_ZAP* and *Kcat_LCKPU_CD3z*. Simulated results are presented for low-antigen concentration (top row) and high-antigen concentration (bottom row). (*a*) Simulated results with optimized *Kcat_ZAP* only, (*b*) simulated results with optimized *Kcat_LCKPU_CD3z* only, and (*c*) simulated results with both *Kcat_ZAP* and *Kcat_LCKPU_CD3z* optimized.

To test the consequences of targeting the parameters identified by the optimization procedure, we again performed simulations with varying antigen concentrations, with one or both of the selected parameters set to their optimized values and all others kept at their default values. Based on our results, setting *Kcat_ZAP* to the optimized value resulted in a reduction of the activation time (figure 6*a*), with a greater fraction of second-generation cells becoming active compared to first-generation cells (table 3). However, setting *Kcat_LCKPU_CD3z* to the optimized value was sufficient to not only induce a drastic reduction in cell activation time, but also to make first-generation cells more efficient than second-generation cells (figure 6*b*,*c* and table 3). Particularly, with *Kcat_LCKPU_CD3z* optimized alone, both populations showed 100% activation with mean activation times 5.2 min versus 6.3 min for first- and second-generation cells, respectively; when both *Kcat_LCKPU_CD3z* and *Kcat_ZAP* were optimized, both populations showed 100% activation with mean activation times of 3.1 min versus 3.5 min for first- and second-generation cells, respectively (table 3). When we reran simulations of kinetic variability with the same optimized parameters, we obtained similar results. Specifically, *Kcat_LCKPU_CD3ζ* optimization is predicted to be sufficient to make first-generation cells respond faster to antigen presence than second-generation cells (figure 7 and table 4).

**Table 3.** Comparison of optimized systems with antigen variability.

CAR	parameter optimized	%-age cells active	mean activation time (s.d.), min
first generation	*Kcat_ZAP* only	99.9	10.0 (3.6)
	*Kcat_LCKPU_CD3z* only	100.0	5.2 (1.3)
	both	100.0	3.1 (0.6)
second generation	*Kcat_ZAP* only	100.0	4.6 (1.2)
	*Kcat_LCKPU_CD3z* only	100.0	6.3 (1.8)
	both	100.0	3.5 (0.8)

**Table 4.** Comparison of optimized systems with kinetic variability.

CAR	parameter optimized	%-age cells active		mean activation time (s.d.), min	
		low antigen	high antigen	low antigen	high antigen
first generation	*Kcat_ZA*P only	92.7	98.0	9.1 (5.5)	5.2 (3.4)
	*Kcat_LCKPU_CD3z* only	98.1	97.2	5.9 (3.4)	6.7 (4.0)
	both	98.9	98.7	3.4 (2.0)	4.0 (2.5)
second generation	*Kcat_ZAP* only	99.0	99.1	3.0 (1.7)	2.4 (1.2)
	*Kcat_LCKPU_CD3z* only	98.9	99.0	2.7 (1.5)	2.9 (1.6)
	both	99.1	99.2	2.0 (1.1)	2.1 (1.1)

## 4. Discussion

In this study, we applied an existing ODE-based mechanistic model of CAR-induced ERK signalling to compare the performance of first- and second-generation engineered CAR T cells, given external and internal sources of variability. By simulating variable antigen concentrations, we showed that costimulatory signalling from CD28 causes both a reduced activation time and more consistent population response. In addition, by simulating stochastic kinetic interaction parameters, we were able to show that when considering kinetic variability caused by biochemical noise, second-generation cells yield similar results: reduced activation time and variability of the population response compared to first-generation cells. In order to track the mechanism by which CD28 induces this change, we repeated simulations of kinetic variability with only one CD28-mediated mechanism available at a time: the association between CD28 and Grb2, the association between CD28 and GADS, or the CD28-mediated change in the catalytic activity of LCK. We found that the effect of CD28 on the catalytic activity of LCK is both necessary and sufficient to produce the performance improvement in second-generation cells. Next, we set out to identify parameters that could be modulated to further reduce response times and variability in the population response. To this end, we trained a GBTE, which can predict system activation time based on values of kinetic parameters. After confirming the predictor's accuracy, we quantified the importance of each parameter in the predictor's estimation of activation time. Using this method, we isolated five influential kinetic parameters. Then, we performed a constrained optimization procedure on our model by using an objective function that sought to minimize system activation time while manipulating as few of the five candidate parameters as possible. Based on the results of this optimization procedure, the most optimal targets are predicted to be the catalytic activity of LCK in phosphorylating ITAM motifs of the CD3*ζ* domain (*Kcat_LCKPU_CD3z*) and the catalytic activity of the kinase ZAP70 (*Kcat_ZAP*). To elucidate how implementing the values suggested by the optimization procedure would affect population performance of the engineered T cells, we repeated simulations of the system with antigen variability and kinetic variability by using the optimized parameter value. Optimizing *Kcat_ZAP* resulted in an overall reduction in cell response time but preserved the overall superiority of second-generation cells. By contrast, manipulating *Kcat_LCKPU_CD3z* independently or in conjunction with *Kcat_ZAP* not only drastically improved performance, but also made first-generation cells perform at least as efficiently as, and in some cases better than, second-generation cells. The suggested manipulations closely align with the central role both enzymes play in T-cell signalling. The Src family kinase LCK is recruited to CD3*ζ* after target recognition and phosphorylates the ITAM motifs, making them available for docking by downstream proteins involved in signal transduction, including ZAP70 [27]. Active ZAP70 associates with phosphorylated ITAMs of CD3*ζ* and phosphorylates different targets, including the adaptor protein LAT. After being phosphorylated by ZAP70, LAT recruits signalling proteins involved multiple cascades, including the ERK cascade [28]. We note that the inputs to the GBTE are the kinetic parameters from our computational model. Though such parameters may be difficult to measure experimentally using existing experimental techniques, we produce insights as to their impact on the population-level response of CAR T cells.

Experimental studies have shown that the second-generation CAR constructs with a CD28 costimulatory domain promote a better immune response during *in vivo* testing, compared to first-generation constructs. While theoretical underpinnings of this phenomenon were explored in prior research, our work provides a new context for this discrepancy. Particularly, we showed that the incorporation of the CD28 costimulatory domain results in both shorter and more consistent response times in the face of various sources of variability. A population of CAR T cells infused into the patient's bloodstream would encounter highly variable external and internal conditions. As this variability is not reflected in the *in vitro* setting, our predictions provide valuable insight into the features of engineered CAR T cells. In addition, we explored potential routes for the further improvement of CAR T-cell therapies. So far, work has mostly focused on incorporating more signalling domains or activity-dependent expression cassettes into the structure of the CAR [2]. We explored the alternative possibility of enhancing CAR T-cell response by manipulating the catalytic activity of enzymes involved in signal transduction. The ability to engineer enzymes with desired properties, including improved catalytic activity, has already found broad applications in biotechnology [29]. While traditional methods use either a targeted substitution of key amino acids or directed evolution of random mutations, new approaches based on machine learning empower specialists to look for candidates *in silico* [30]. Based on our model, engineering a more catalytically active isoform of LCK would cause first-generation cells to become at least as efficient as otherwise equivalent CD28-bearing CAR T cells. It is established that despite their greater efficacy, second-generation cells suffer from multiple side effects, and mitigating those side effects is a significant concern. We believe that enhancing LCK catalytic activity as an alternative to having the CD28 domain in the CAR structure can be one such mitigating strategy, since it would potentially exclude undesirable effects of CD28 without compromising ERK signalling efficacy.

Along with the significant findings produced by our work, we recognize some limitations of our approach that can be addressed in the future. When exploring the population response of CAR T cells to stochastic antigen concentrations, we assumed lognormal distribution parameters chosen to make the distribution fall within an experimentally observed range. For simulations of kinetic heterogeneity, we assumed a normal distribution centred around the accepted default value with a standard deviation as a third of this value. However, we have no unequivocal evidence that the assumed distributions in fact reflect what is observed experimentally. While our assumed distributions have a clear experimental basis both in terms of the distribution chosen and the parameters, they are not the only option possible. By trying other assumed distributions via altering the distribution parameters, a more comprehensive understanding of the CAR T-cell's population response may emerge. Another limitation of our study concerns the choice of optimized values for *Kcat_ZAP* and *Kcat_LCKPU_CD3z* when considering heterogeneity. Our objective function penalized changes in each parameter proportional to the absolute value of the logarithm of the fold change compared to its baseline value. Since the baseline value of *Kcat_LCKPU_CD3z* is different between first- and second-generation cells owing to CD28's effects in the latter, we obtained different optimal values of *Kcat_LCKPU_CD3z* for first- and second-generation cells. However, since our goal was to simulate the observed effects of an artificially enhanced LCK, we assumed that the catalytic properties of such enhanced LCK would be the same regardless of the CAR structure. Thus, we were compelled to use the same optimal *Kcat_LCKPU_CD3z* value when performing simulations that accounted for antigen and/or kinetic variability. Future experimental work can explore the validity of our assumption.

## 5. Conclusion

Our work focused on exploring the difference in ERK activation times between engineered CAR T cells with or without CD28 under various sources of cellular variability. We discovered that in line with expectations, the CD28 domain increases the proportion of cells that become activated based on ERK phosphorylation. Further, the model increases our mechanistic understanding of the role of CD28, predicting that CD28 confers shorter and more consistent activation times across the cell population. In addition, we discovered that the catalytic activity of LCK can serve as a valid target for the further improvement of CAR T-cell activation, since increasing the value of the corresponding parameter causes a pronounced improvement in the performance of first-generation CAR T cells. Our work provides novel quantitative insights that can guide the design of CAR-engineered cells for immunotherapy.

## Data Availability

Data and relevant code for this research work are stored in GitHub: https://github.com/FinleyLabUSC/CAR-T-Signaling-in-variability and have been archived within the Zenodo repository: https://zenodo.org/record/6828897. Data are also provided in the electronic supplementary material [31].
